# Radioiodination of Aryl-Alkyl Cyclic Sulfates

**DOI:** 10.3390/molecules171113266

**Published:** 2012-11-07

**Authors:** Chandra Mushti, Mikhail I. Papisov

**Affiliations:** 1 Massachusetts General Hospital, Harvard Medical School and Shriners Hospital for Children, Shriners Hospital for Children—Boston, Room 439, 51 Blossom St., Boston, MA 02114, USA; 2 Massachusetts General Hospital, Harvard Medical School and Shriners Hospitals for Children, Shriners Hospital for Children—Boston, Room 224, 51 Blossom St., Boston, MA 02114, USA

**Keywords:** iodine, cyclic sulfate, Positron Emission Tomography, ^124^I, enzyme substrate

## Abstract

Among the currently available positron emitters suitable for Positron Emission Tomography (PET), ^124^I has the longest physical half-life (4.2 days). The long half-life and well-investigated behavior of iodine *in vivo* makes ^124^I very attractive for pharmacological studies. In this communication, we describe a simple yet effective method for the synthesis of novel ^124^I labeled compounds intended for PET imaging of arylsulfatase activity *in vivo*. Arylsulfatases have important biological functions, and genetic deficiencies of such functions require pharmacological replacement, the efficacy of which must be properly and non-invasively evaluated. These enzymes, even though their natural substrates are mostly of aliphatic nature, hydrolyze phenolic sulfates to phenol and sulfuric acid. The availability of [^124^I]iodinated substrates is expected to provide a PET-based method for measuring their activity *in vivo*. The currently available methods of synthesis of iodinated arylsulfates usually require either introducing of a protected sulfate ester early in the synthesis or introduction of sulfate group at the end of synthesis in a separate step. The described method gives the desired product in one step from an aryl-alkyl cyclic sulfate. When treated with iodide, the source cyclic sulfate opens with substitution of iodide at the alkyl center and gives the desired arylsulfate monoester.

## 1. Introduction

Arylsulfatases [1] are a group of lysosomal enzymes responsible for hydrolyzing the sulfate ester group esters in various biological substrates. Substrates for arylsulfatases can be as simple as steroid sulfates and as complex as glycosaminoglycans [1,2]. Deficiency or reduced activity of these enzymes lead to the accumulation of sulfated molecules involved in important cellular functions involved in hormone regulation, degradation, and signaling pathways [3,4,5].

Development of enzyme replacement therapies is presently hindered by the lack of suitable non-invasive methods for assessment of the activity of the enzyme of interest in the organs and tissues. Recently, we conducted several preclinical studies where radiolabeled enzyme molecules were studied by Positron Emission Tomography (PET) to determine the kinetics of their transfer from the injection point to the organs of interest [6,7]. Such studies give quantitative information on the delivery of the enzyme molecules to the affected tissues and the initial deposition [8], but they don’t provide information on the enzyme activity in these tissues. Development of non-invasive methods suitable for direct measurement of enzyme activity in preclinical models, as well as in a clinical setting, would shorten the drug development cycle and improve the assessment of the efficacy of the therapy. 

We have investigated the pharmacokinetics of arylsulfatase A (ARSA) in rodents [7] and non-human primates [9], which provided the pharmacokinetics data necessary for estimating the initial enzyme deposition in the tissue as a function of administration modality. Presently, we are developing a PET-based method enabling the assessment of enzyme activity, rather than measuring the amount of protein. The method will utilize arylsulfatase substrates modified with a label that is detectable by imaging and, upon enzymatic reaction, giving products that are retained in the tissues where the reaction has occurred longer than the respective substrates would be retained. The products of the enzymatic reaction will thus “label” the tissues where the enzyme activity is present. Considering the quantitative character of PET, we focus on substrates labeled with positron emitting ^124^I. Iodine-124 is used here as a positron emitter with a physical half-life of 4.2 days, which enables non-invasive quantitative tracking of the administered radioactivity *in vivo* for several days [10]. The above approach suggests developing a method for synthesizing radiolabeled arylsulfatase substrates that undergo enzymatic hydrolysis without affecting the enzyme activity. 

**Figure 1:**
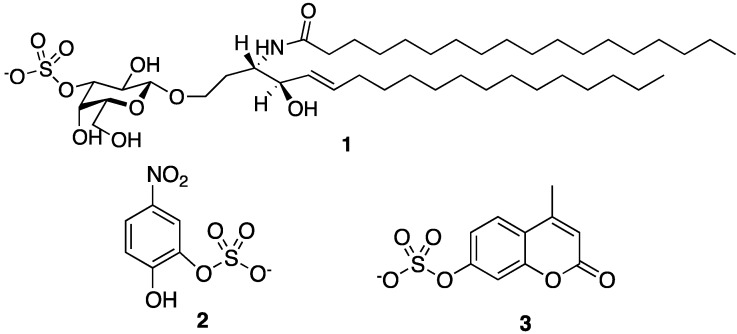
Natural and known phenolic substrates of ARSA.

The natural substrates for ARSA are sulfosphingolipids [11] (Figure 1, **1**). Such compounds form micelles and thus are unlikely suitable as molecular probes for arylsulfatase activity *in vivo* because of their incorporation in the lipid compartment *in vivo*, which is not compatible with the proposed imaging approach. Synthetic substrates, e.g., 4-nitrocatechol sulfate and 4-methylumbelliferyl sulfate (Figure 1, **2** and **3**), are known to be hydrolyzed by ARSA (as well as other arylsulfatases) under physiological conditions. They give colored and fluorescent products upon desulfation [12], respectively, which enables their use in *in vitro* assays of arylsulfatase activity. While our approach suggests using similar ^124^I-labeled structures that can be hydrolyzed by arylsulfatases, the modality of their *in vivo* use suggests designing molecules which, after desulfation, can bind tissue components (e.g., via hydrophobic interactions) and thus, “label” the tissues with ^124^I. 

One of the major hurdles in designing compounds suitable for use as substrates for arylsulfatases is the introduction of the sulfate ester. This group is sensitive to acidic conditions and is known to undergo nucleophilic substitution when present on an alkyl scaffold. The currently available procedures usually utilize either the introduction of sulfate ester at the end of the synthetic sequence, or introducing a protected sulfate ester at the beginning, followed by removal of the protection at the end of the sequence. 

Although these methods are attractive for the synthesis of sulfate esters of stable compounds, they are not optimal for radiochemical syntheses. The latter require handling of the radiolabeled precursors through multiple chemical transformations, which not only generates radioactive waste but also leads to the loss of radioactivity due to the radionuclide decay. For the PET imaging applications, radiolabeling should be, ideally, the last stage of the process so that the radiolabeled product would be used for the imaging immediately after the labeling and fast purification. Here, we report a new method for synthesis of radioiodinated arylsulfatase substrates, where the introduction of the radiolabel (^124^I) and formation of the sulfate ester are achieved in one step.

## 2. Results and Discussion

Cyclic sulfates [13] are diesters of sulfuric acid, commonly used in organic chemistry for mono functionalization of vicinal diols. They are better leaving groups than the more commonly used leaving groups tosylates and mesylates, and are fairly stable compounds [14]. We speculated that, if we could prepare an asymmetrical cyclic sulfate involving a phenol and an aliphatic alcohol, a nucleophilic reagent would react with the aliphatic side of the cyclic sulfate and generate a phenolic sulfate ester on the other side. 

**Scheme 1:**
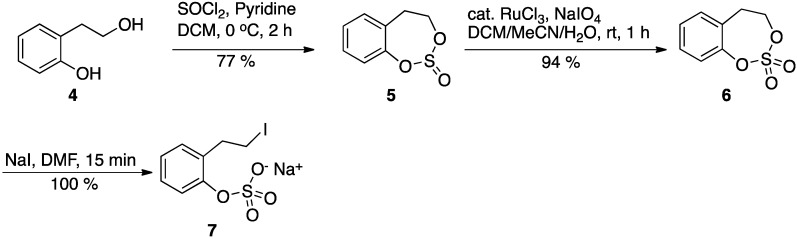
Synthesis and ring opening of cyclic sulfate **6**.

We tested our hypothesis using a commercially available 2-hydroxyphenethyl alcohol (**4**), which, when treated with thionyl chloride and pyridine at 0 °C in anhydrous dichloromethane, gave cyclic sulfite **5** in excellent yield. This cyclic sulfite was then oxidized to the desired cyclic sulfate **6** quantitatively using catalytic RuCl_3_ and sodium periodate. Treatment of the cyclic sulfate with sodium iodide in DMF gave the sulfate ester **7** in quantitative yield, as shown in Scheme 1. We note that primary iodides such as the model compound (**7**) may be insufficiently stable *in vivo* for imaging purposes. The method, however, is intended for developing secondary iodides (as described below) and, eventually, [18F]fluorides that are significantly more stable. 

**Scheme 2:**
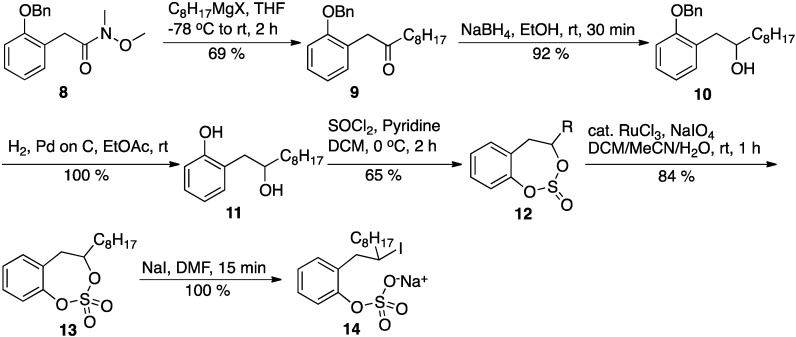
Synthesis and ring opening of cyclic sulfate **13**.

To extend the versatility of this method, we synthesized a cyclic sulfate with a long aliphatic side chain as shown Scheme 2. The synthesis commenced from the amide **8**, synthesized in approximately 50% yield according to a known three-step procedure [15] from commercially available 2-hydroxy-phenylacetic acid. The amide **8** was then treated with the commercially available octylmagnesium bromide solution to give the ketone derivative **9**. Ketone **9** was reduced to alcohol **10** with sodium borohydride. The subsequent removal of benzyl protection, using catalytic 10% Pd on C under a hydrogen balloon, gave the substituted 2-hydroxyphenethyl alcohol **11**. This compound was converted to the desired cyclic sulfate **13** under the same conditions as described in Scheme 1. 

Labeling of **6** with [^124^I]NaI was accomplished using the same procedure as described in Scheme 1. The [^124^I] sodium iodide for this purpose was obtained from IBA Molecular (Richmond, VA, USA) as a carrier free aqueous solution in 0.02 M NaOH, 20–50 mCi/mL. Water was removed by azeotroping with anhydrous acetonitrile under an argon stream. 

After the removal of water, a solution of cyclic sulfate in anhydrous DMF (100 μL, 1 mg/mL to 0.5 μCi of NaI) was added to the solid [^124^I] sodium iodide and kept at ambient temperature for about 30 min. The resulting product was then purified by reverse phase HPLC using UV and gamma radiation detector. From our studies on **6**, we found that the presence of significant amounts of water drastically slows down the reaction. 

During the course of our studies on ring opening of cyclic sulfate with NaI, we found that adding an aqueous solution of NaI to a DMF solution of the cyclic sulfate led to a significant slowdown of the reaction; the latter was not complete after 24 h at ambient temperature. Similarly, the reaction was slowed significantly when carried out in the polar protic solvents, e.g., ethanol. In acetone, the reaction led to the formation of an uncharacterized non-polar product. Thus, without the need to be anhydrous, DMF was found to be the best solvent for the reaction.

This methodology describes the synthesis of new aryl-alkyl cyclic sulfate, which can serve as intermediates in organic synthesis. The method described here offers another significant advantage for synthesizing substrates for arylsulfatase A because it is applicable not only to ^124^I, but it can also be easily adopted by other PET-compatible radionuclides, such as F^18^ and C^11^. 

## 3. Experimental

### General

All reactions were performed under an atmosphere of argon in oven dried glassware using anhydrous solvents on 4 Å molecular sieves obtained from Acros Organics/Thermo Fisher Scientific (Pittsburgh, PA, USA). Solvents were always handled under argon atmosphere. The ambient temperature was set at 21 °C. 

Yields refer to chromatographically and spectroscopically homogenous materials, unless otherwise stated. Reactions were monitored by thin-layer chromatography (TLC) carried out on 0.25 mm E. Merck silica gel plates (60F-254), using UV light as a visualizing agent, and an aqueous solution of basic potassium permanganate and heat as developing agents. Purifications were done on a Biotage flash purification system using pre-packed Biotage SNAP columns. 

Products were characterized by ^1^H-NMR/^13^C-NMR on either a Varian 500 MHz, or 400 MHz spectrometer in a deuterated solvent using tetramethylsilane (TMS) as an internal standard. Chemical shifts are given in δ (ppm) and *J*-values in Hz. High-pressure liquid chromatography (HPLC) was performed using Waters 1525 binary HPLC pump and Waters 2489 UV/Visible detector operating at 254 nm on a Waters symmetry C18 3.5 μm, 4.6/75 mm column. MeOH HPLC solvent was obtained from Sigma-Aldrich (St. Louis, MO, USA). 

*4,5-Dihydrobenzo[d][1,3,2]dioxathiepine 2,2-dioxide* (**6**): Anhydrous pyridine (3.4 mL, 41.62 mmol) was added to a solution of the 2-phenethanol **4** (2.3 g, 16.65 mmol) in anhydrous DCM (70 mL), and the mixture was cooled to 0 °C in an ice bath. Then, freshly distilled thionyl chloride (1.22 mL, 16.65 mmol) was added to the above solution. The resultant reaction mixture was then stirred for 2 h with gradual warming to the ambient temperature. At this point, TLC of the reaction mixture indicated complete consumption of the starting material. The reaction mixture was then diluted with more DCM, transferred to a separatory funnel, and washed with cold dilute HCl twice. Then the DCM layer was washed with water (2×) and brine (1×), and, finally, the DCM layer was dried with anhydrous sodium sulfate and concentrated to give the cyclic sulfite **5**, 2.8 g, as an oily liquid (Rf: 0.58; 20% EtOAc/hexane). The product was used in the subsequent step without purification.

Catalytic ruthenium chloride (0.02 mol %, 63 mg) was added to the crude cyclic sulfite **5** (2.8 g, 15.2 mmol), in acetonitrile (40 mL) and DCM (40 mL). Then, a solution of sodium periodate (4.9 g, 22.8 mmol) in water (60 mL) was slowly added dropwise to the above solution at room ambient temperature. The reaction mixture was then stirred at room temperature until all of the starting material was consumed (about 1 h). All of the volatile solvents were removed in vacuo and the aqueous layer was extracted with EtOAc (3×). The combined ethyl acetate layer was washed with saturated sodium bicarbonate solution (2×), water (2×), and brine (1×). Finally, the organic layer was dried over anhydrous sodium sulfate, concentrated, and chromatographed (hexane/EtOAc 5–50%) over silica gel to give pure cyclic sulfate (**6**), 2.9 g, 87%, as white solid. Rf: 0.28 (EtOAc/hexane). ^1^H-NMR (CDCl_3_; 500 MHz) δ 7.35 (t, 7.5 Hz, 1 H), 7.26 (dd, 13.5 Hz, 7,5 Hz, 2 H), 7.19 (d, 7.5 Hz, 1 H), 4.64 (m, 2 H), 3.26 (m, 2 H); ^13^C-NMR (CDCl_3_, 125 MHz) δ 149.9, 131.1, 130.2, 129.5, 127.7, 121.9, 72.6, 33.6; HPLC: R_T_ 9.1 min on Symmetry C18 column from Waters, flow 1 mL/min, linear gradient 5 to 95% MeOH/Water.

*Sodium 2-(2-Iodoethyl)phenyl sulfate* (**7**): Solid sodium iodide (13 mg, 0.088 mmol) was added to a solution of cyclic sulfate **6** (17.5 mg, 0.088 mmol) in the DMF (0.35 mL) at ambient temperature. The reaction was monitored by the TLC to document the consumption of the starting material. Within 15 min, all of the starting material was consumed. The DMF was removed *in vacuo* and the residue was dried thoroughly *in vacuo* at ambient temperature to give the pure product **7**, yield 100%; ^1^H-NMR (DMF-d_7_, 400 MHz) δ 7.21 (m, 3 H), δ 7.01 (dt, 1 Hz, 8 Hz, 1 H), 3.55 (m, 2 H), 3.26 (m, 2 H); 13C-NMR (100 MHz, DMF-d_7_) δ 152.2, 133.2, 130.3, 127.7, 123.6, 122.3, 35.9, 6.5; HPLC: R_T_ 6.1 min on Symmetry C18 column from Waters, flow 1 mL/min, linear gradient 5 to 95% MeOH/Water.

*1-(2-(Benzyloxy)phenyl)decan-2-one* (**9**): A solution of amide **8** (570 mg, 2 mmol) in anhydrous THF (8 mL) was cooled to −78 °C in a dry ice-acetone bath under an argon atmosphere. To this cooled solution, a solution of octylmagnesium bromide (2 M, 1.25 mL, 2.5 mmol) in ether was added. After the addition was complete, the reaction was allowed to proceed for 2 h with gradual warming to the ambient temperature. The reaction was quenched by the addition of a saturated ammonium chloride solution and the THF was removed *in vacuo*. The reaction mixture was transferred to a separatory funnel and extracted with diethyl ether (3×). The combined organic layer was washed with water (1×) and brine (1×), dried over anhydrous sodium sulfate, concentrated and chromatographed on silica gel to give pure ketone **9**, 415 mg, 61%, as viscous oil. R_f_: 0.77 (15% EtOAc in hexane); ^1^H-NMR (CDCl_3_, 500 MHz) δ 7.39 (m, 5 H), 7.34 (m, 1 H), 7.25 (m, 1 H), 7.16 (m, 1 H), 6.96 (m, 1 H), 5.06 (s, 2 H), 3.71 (s, 2 H), 2.4 (t, 7 Hz, 2 H), 1.52 (m, 2 H), 1.23 (bm, 10 H), 0.89 (t, 7.5 Hz, 3 H); ^13^C-NMR (125 MHz, CDCl_3_) δ 206.8, 156.4, 136.9, 131.3, 128.5, 128.3, 127.8, 127.3, 124.1, 120.8, 111.6, 69.9, 44.7, 42.2, 31.8, 29.3, 29.1, 29.09, 23.7, 22.6, 14.0.

*1-(2-(Benzyloxy)phenyl)decan-2-ol* (**10**): To a solution of ketone **9** (415 mg, 1.23 mmol) in absolute ethanol (5 mL), solid sodium borohydride (93 mg, 2.46 mmol) was added at ambient temperature. The reaction mixture was stirred at ambient temperature for 30 min. At this point, the TLC indicated complete consumption of the starting material, and the reaction was quenched by the addition of a saturated ammonium chloride solution. All of the ethanol was removed *in vacuo*, and the residue was transferred to a separatory funnel and extracted with EtOAc (3×). The combined organic layer was washed with water (2×) and brine (1×). Finally, the organic layer was dried over anhydrous sodium sulfate, concentrated, and chromatographed on silica gel to give pure alcohol **10**, 393 mg, 94%, as viscous oil. Rf: 0.48 (10% EtOAc in hexane); ^1^H-NMR (500 MHz, CDCl_3_) δ 7.41 (m, 5 H), 7.20 (m, 2 H), 6,94 (m, 2 H), 5.09 (s, 2 H), 3.89 (bs, 1 H), 3.00 (dd, 4 Hz, 14 Hz, 1 H), 2.68 (dd, 8 Hz, 14 Hz, 1 H), 1.93 (bs, 1 H), 1.50 (m, 2 H), 1.27 (bm, 12 H), 0.89 (t, 7 Hz, 3 H); ^13^C-NMR (125 MHz, CDCl_3_) δ 156.7, 136.9, 131.5, 128.5, 127.9, 127.7, 127.5, 127.2, 120.9, 111.7, 71.8, 70.0, 38.8, 37.2, 31.8, 29.7, 29.6, 29.2, 25.7, 22.6, 14.1.

*2-(2-Hydroxydecyl)phenol* (**11**): Catalytic 10% palladium on carbon (20 mg) was added to a solution of alcohol **10** (393 mg, 1.15 mmol) in EtOAc (4 mL). Then the reaction flask was purged with hydrogen (by applying vacuum and flushing hydrogen gas). This process was repeated three times, and then the reaction proceeded overnight under a balloon of hydrogen gas. The reaction mixture was then filtered through a pad of Celite, and the Celite pad was washed thoroughly with EtOAc. Finally, EtOAc was removed *in vacuo* to give the desired product **11**—a pale yellow oil (285 mg, 99%). Thus, the product obtained was sufficiently pure for use in the next step; an analytically pure sample for NMR was obtained by chromatographing a small sample on silica gel. Rf: 0.45 (25% EtOAc in hexane); ^1^H-NMR (500 MHz, CDCl_3_) δ 8.19 (bs, 1 H), 7.15 (m, 1 H), 7.02 (m, 1 H), 6.91 (m, 1 H), 6.84 (m, 1 H), 4.00 (m, 1 H), 2.85 (dd, 2 Hz, 15 Hz, 1 H), 2.80 (dd, 7 Hz, 15 Hz, 1 H), 2.39 (bs, 1 H), 1.53 (m, 1 H), 1.28 (m, 12 H), 0.88 (t, 7 Hz, 3 H); ^13^C-NMR (125 MHz, CDCl_3_) δ 155.9, 131.5, 128.3, 125.9, 120.2, 117.2, 74.6, 40.3, 38.9, 36.9, 31.8, 29.5, 29.4, 25.6, 22.6, 14.1.

*4-Octyl-4,5-dihydrobenzo[d][1,3,2]dioxathiepine 2,2-dioxide* (**13**): Anhydrous pyridine (330 μL, 3.6 mmol) was added to a solution of the substituted 2-phenethanol **11** (340 mg, 1.44 mmol) in anhydrous DCM (6 mL), and the mixture was cooled to 0 °C in an ice bath. Then, freshly distilled thionyl chloride (115 μL, 1.58 mmol) was added to this solution. The reaction mixture was then removed from the ice bath and stirred for 2 h while warming up to the ambient temperature. At this point, the TLC of the reaction mixture indicated the complete consumption of the starting material. The reaction mixture was diluted with more DCM, transferred to separatory funnel, and washed with cold dilute HCl (2×). Then, the DCM layer was washed with water (2×) and brine (1×). Finally, the DCM layer was dried over anhydrous sodium sulfate and concentrated to give the cyclic sulfite **12** (360 mg, 87%) as an oily liquid. The product was then used in the next step without purification. The product formed two spots on the TLC (Rf: 0.80 & 0.72; 10% EtOAc/hexane) due to the formation of diastereomers.

Catalytic ruthenium chloride (0.02 mol %; 5 mg) was added to the solution of the crude cyclic sulfite **12** (350 mg, 1.20 mmol) in acetonitrile (4 mL) and DCM (4 mL). Then, a solution of sodium periodate (380 mg, 1.77 mmol) in water (6 mL) was added to this solution dropwise at ambient temperature. The reaction mixture was then stirred at ambient temperature until the starting material was consumed (about 1 h). All of the volatile solvents were removed *in vacuo* and the aqueous layer was extracted with EtOAc (3×). The combined ethyl acetate layer was washed with saturated sodium bicarbonate solution (2×), water (2×), and brine (1×). Finally, the organic layer was dried over anhydrous sodium sulfate, concentrated, and chromatographed (hexane/EtOAc 5–15%) on silica gel to give pure cyclic sulfate **13** (288 mg, 78%), as a pale yellow, viscous oil. Rf: 0.43 (10% EtOAc in hexane); ^1^H-NMR (500 MHz, CDCl_3_) δ 7.34 (m, 1 H), 7.25 (m, 2 H), 7.16 (m, 1 H), 4.80 (m, 1 H), 3.41 (dd, 9.5 Hz, 16 Hz, 1 H), 2.90 (d, 16 Hz, 1 H), 1.89 (m, 1 H), 1.73 (m, 1 H), 1.29 (m, 12 H), 0.88 (t, 7 Hz, 3 H); ^13^C-NMR (125 MHz, CDCl_3_) δ 150. 0, 131.2, 129.4, 127.6, 126.5, 86.2, 40.3, 38.9, 35.0, 31.8, 29.1, 29.0, 24.9, 22.6, 14.1; HPLC: R_T_ 9.4 min, flow 1 mL/min, gradient 10 to 100% MeOH/Water.

*Sodium 2-(2-iododecyl)phenyl sulfate*: Solid sodium iodide (15 mg, 0.1 mmol) was added to a solution of cyclic sulfate (**13**, 26 mg, 0.083 mmol) in the DMF (0.5 mL) at ambient temperature. The reaction was monitored by TLC to document the consumption of the starting material. Within 15 min, the latter was consumed, and the DMF was removed *in vacuo*. The residue was dried thoroughly *in vacuo* at ambient temperature to give a pure product (yield 100%); ^1^H-NMR (DMF-d_7_, 400 MHz) δ 7.56 (dd, 1.2 Hz, 8 Hz, 1 H), 7.20 (m, 2 H), 7.00 (dt, 1.2 Hz, 8 Hz, 1 H), 3.79 (bs, 1 H), 3.40 (dd, 7 Hz, 14 Hz, 1 H), 3.33 (dd, 8 Hz, 14 Hz, 1 H), 1.73 (m, 2 H), 1.26 (m, 12 H), 0.86 (t, 6.8 Hz, 3 H); ^13^C-NMR (DMF-d_7_, 100 MHz) δ 153.1, 132.5, 131.0, 127.7, 123.3, 122.1, 43.4, 40.9, 40.2, 32.2, 30.0, 29.8, 29.6, 29.1, 22.9, 14.2; HPLC: R_T_ 8.1 min, flow 1 mL/min, gradient 10 to 100% MeOH/Water.

*Procedure for Labeling 6 with ^124^I*: 200 μL of anhydrous MeCN were added to a solution of [^124^I]NaI (500 μCi) in 15 μL 0.02 M NaOH, and the solvents were evaporated under steady stream of argon. After the solvent was completely removed, an extra 100 μL of MeCN was added, and the solvent was removed again, as above. This procedure was repeated two more times to remove most of the water from [^124^I]NaI. Then, a solution of cyclic sulfate (100 μL, ~1 mg/mL) in DMF was added to the residue, and the iodination reaction was allowed to proceed for 30 min. Then, the reaction mixture was analyzed by HPLC using dual detection (UV and gamma) to distinguish the desired product from the unreacted [^124^I]iodide. Under the HPLC conditions (see above), the [^124^I]iodide had an R_T_ of 1 min and the desired compound eluted at 7.3 min, as observed on gamma-detector. Retention times for the labeled compound on gamma-detector (7.3 min), and unlabeled compound on UV detector (6.1 min), are different because the gamma-detector was externally connected.

## 4. Conclusions

We developed a very effective method for labeling synthetic arylsulfatase substrates with ^124^I. In this method, the steps of introducing the sulfate ester and radioactive iodine converge into one stage. This not only simplifies the synthesis, but also reduces the time between the radionuclide production and use, which preserves the radioactivity. We expect that the method can be easily adopted for labeling arylsulfates with other radionuclides suitable for PET, such as F^18^ and C^11^.

## Supplementary Materials

Supplementary materials can be accessed at: http://www.mdpi.com/1420-3049/17/11/13266/s1.
