# Supplementary material for: Radioiodination of Aryl-Alkyl Cyclic Sulfates
Source: Molecules. 2012 Nov 7;17(11):13266–74. doi: 10.3390/molecules171113266 (PMC4422485; doi:10.3390/molecules171113266)

Supplementary Information

Figure S1. Iodination of cyclic sulfoester **6** with formation of iodide **7**. Reaction mixture HPLC, UV detection (see text).

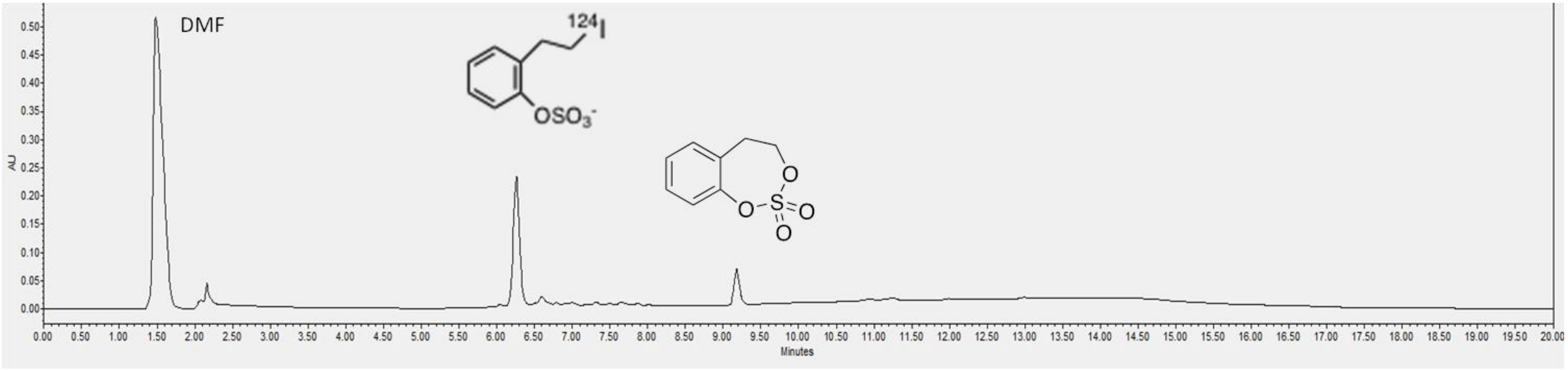

Figure S2. Radioiodination of the cyclic sulfoester **6** with formation of radioactive iodide [^{124}I]**7**. Reaction mixture HPLC, gamma detection (see text). Note that the low (subpicomolar) concentrations result in peak broadening as compared to the HPLC trace in the Figure S1.

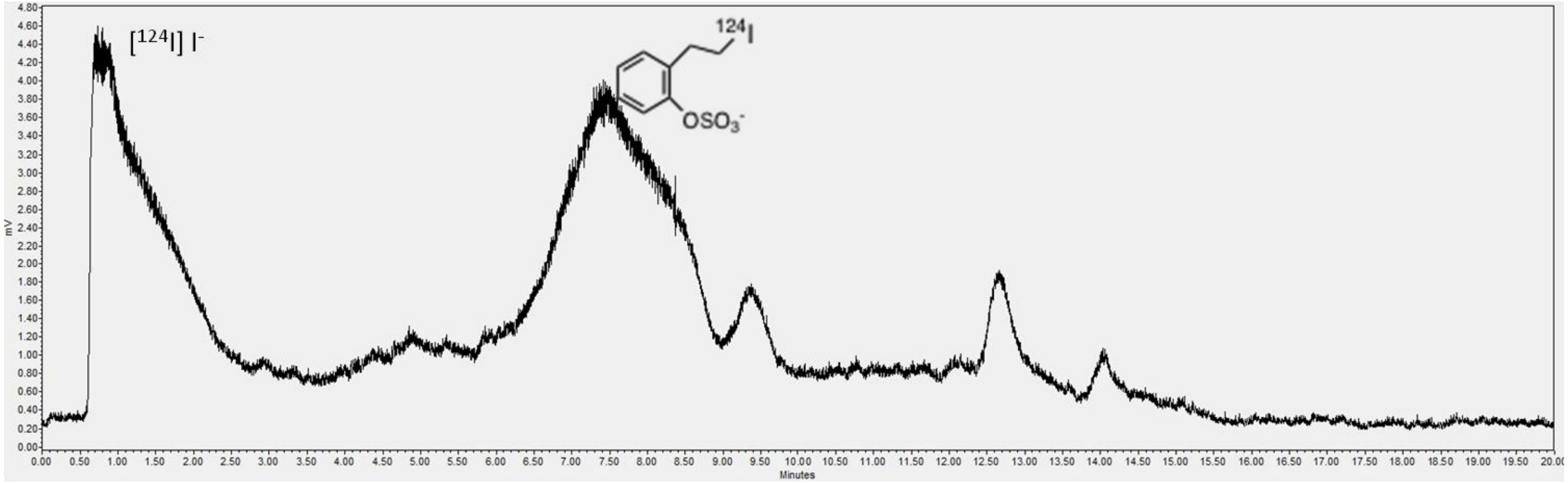

Supplement: Supplementary file 1 [file molecules-17-13266-s001.pdf]
